# Barkhausen Noise in 100Cr6 Bearing Steel as a Function of Microstructure and Stress State

**DOI:** 10.3390/ma19143135

**Published:** 2026-07-21

**Authors:** Martin Pitoňák, Anna Mičietová, Ján Moravec, Miroslav Neslušan, Štefan Toth, Branislav Mičieta

**Affiliations:** 1 Faculty of Civil Engineering, University of Žilina, Univerzitná 1, 01026 Žilina, Slovakia; martin.pitonak@uniza.sk; 2 Faculty of Mechanical Engineering, University of Žilina, Univerzitná 1, 01026 Žilina, Slovakia; anna.micietova@fstroj.uniza.sk (A.M.); jan.moravec@fstroj.uniza.sk (J.M.); branislav.micieta@fstroj.uniza.sk (B.M.); 3 Faculty of Management Science and Informatics, University of Žilina, Univerzitná 1, 01026 Žilina, Slovakia; stefan.toth@fri.uniza.sk

**Keywords:** grinding, stress state, microstructure, Barkhausen noise, infeed rate, 100Cr6 bearing steel

## Abstract

This study is focused on the unwrapping contribution of microstructure (mainly expressed in terms of dislocation density) and stress state in the quenched bearing steel 100Cr6. Microstructure as well as residual stress state alterations are developed only as a function of variable infeed rates in the flat plunge grinding (other grinding conditions are kept constant). The study is also dealing with the synergistic contribution of residual stress state and the superimposing elastic external stress developed during bending. It was found that the Barkhausen noise after grinding is mostly a function of the thermal softening, whereas the role of residual stress state is only minor. The growing Barkhausen noise emission at the lower infeed rates is connected with the compressive stress, and the tensile stresses are developed at the higher removal rates only. The study also demonstrates good sensitivity of Barkhausen noise when this emission is descending along the compressive external stresses and the ascending evolution along the tensile stresses when the magnetic field is altering along the direction of exerted stress. On the other hand, this evolution is reversed when the altering magnetic field is altered along the transversal direction.

## 1. Introduction

Bearing rings after quenching and tempering are subjected to the grinding cycles in order to achieve the required precision, shape profile and surface roughness. Grinding is generally considered a risky process due to the high cutting speeds and the corresponding heat generated during this process. The major fraction of this heat penetrates to components [1,2], which might initiate unfavourable thermal damage of the surface [3,4], together with the presence of tensile stresses in the surface as well as sub-surface regions [4,5]. Thermal softening as well as tensile residual stresses are the major aspects considered in the early crack initiation and premature failures of bearings in operation. For this reason, the rings of bearing (especially their raceways) should be monitored by a suitable non-destructive technique in order to avoid bearing replacement, which might be quite costly and time-consuming, especially in the wind power industry.

Magnetic Barkhausen noise (MBN) is quite frequently employed as a technique adopted for this task due to the high sensitivity of this technique for such a purpose [3,6]. MBN is a physical phenomenon when a ferromagnetic body is exposed to an alternating magnetic field. This field tends to align domains as well as the corresponding domain walls (DWs) in the direction of this field. However, the process of especially DWs realignment is not continuous since DWs are pinned in their positions and their discontinuous motion in the form of jumps occurs [7,8]. DW jumps produce electromagnetic pulses which can be detected on the surface. Good sensitivity of the MBN technique against the thermal damage in grinding is linked with the reduced pinning strength of the thermally softened structure (the decreased dislocation density and alterations of carbides) with respect to DWs mobility [3,6], as well as considered realignment of DWs due to the presence of tensile stresses [9,10,11]. Their synergistic contribution might be considered. However, the contribution of residual stress in the bearing steels of a quite complicated microstructure containing a high fraction of precipitates (carbides) seems to be overrated. It was clearly reported that despite the high amplitude of the tensile stresses, no visible DW realignment can be observed in 100Cr6 after severe grinding [12]. For this reason, the dislocation density and the altered carbide size, as well as their density, usually prevail over the contribution of the residual stress state. Also, Neel [13] in his strain field theory reported that the fluctuation of spontaneous magnetic polarisation in Fe alloys is mostly linked with the presence of precipitates, and the influence of stresses should be considered mostly in pure Fe or Fe alloys containing a very low fraction of precipitates. On the other hand, the sensitivity of MBN against external load for the conventional soft steels (compressive or tensile stresses) is quite good, despite early saturation being found in some cases [11,14].

Grinding burn detection by the use of MBN is widely investigated due to its high industrial relevance. Some studies correlated MBN with residual stress state [4,6], and the role of microstructure is noted quite briefly. The valuable contribution of carbide dissolution and the corresponding dislocation density alterations with respect to MBN were reported for the case-carburised bearing steels [3,12]. On the other hand, bearing steels containing large Cr rich carbides exhibit increasing MBN together with the growing tensile stresses along grinding wheel wear as well as the altered microhardness [12]. Kuroiwa et al. [15] proposed the machine learning-based prediction of surface roughness and Barkhausen noise in cylindrical grinding with reduced sensor sets. Santa-aho et al. [16] are dealing with the stability of the MBN response with respect to grinding burn detection. Lötfering et al. [17] investigated the conceptual approach for surface integrity evaluation based on MBN. A certain problem with respect to the stress role in MBN can be found in bearing curvature. Tensile stress realigns DWs and, therefore, makes stronger MBN in the direction of this stress at the expense of the perpendicular one. However, valuable differences in MBN are also resulting from the different components’ geometry and their curvature as well (especially in the case of raceways). Therefore, investigation of this aspect on the flat surface eliminates this problem. Furthermore, it should be distinguished between residual stress state as well as external elastic stress and the corresponding magnetostriction (affecting the rate of change in MBN with stress). For these reasons, this study investigates these aspects in the complexity of terms when the stress state is mixed with the altered microstructure (mainly dislocation density) developed under the variable infeed rates.

## 2. Materials and Methods

The experiments were carried out on the bearing steel 100Cr6, quenched and tempered to a hardness of 62 ± 1 HRC. Heat treatment of the samples was carried out in industrial conditions. Samples of the size 200 × 20 × 3.5 mm were quenched from the austenitising temperature 840 °C in the oil at a temperature of 60 °C and tempered for 2 h afterwards at a temperature of 160 °C. The flat infeed grinding process was carried out at 3 different infeed rates and grinding conditions as follows:

- Grinding wheel dressing by the single crystal diamond dresser, dressing infeed rate *a_ed_* = 20 μm, dressing cutting speed *v_cd_* = 25 m·s^−1^, and dressing feed rate *v_fd_* = 90 mm·min^−1^;
- Infeed rates *a_p_* = 0.01, 0.02 and 0.03 mm, feed rate *v_p_* = 12 m·s^−1^, and cutting speed *v_c_* = 25 m·s^−1^;
- Dry grinding; grinding wheel (200 × 25 × 64, A99 60 J9V); grinding machine BPH 20 (TOS, Varnsdorf, Czech Republic);
- Grinding stock 0.3 mm, and 3 spark-out cycles.

Three samples were ground for each of the grinding conditions. The samples after grinding were routinely demagnetised in the vanishing magnetic field, followed by the non-destructive MBN measurement using RollScan 350 (Stresstech Oy, Vaajakoski, Finland). The magnetising frequency 175 Hz of the sine profile and the voltage ± 10 V (magnetising field ± 7.16 kA·m^−1^) were optimised by the use of the frequency and voltage sweeps [18]. The MBN signals were acquired by MicroScan software 5.4.1. The received MBN signals were filtered by the band pass filter 20–1000 kHz and post-processed in order to extract MBN envelopes and further MBN features (*PP*). *PP* represents the position of the MBN envelope maxima in the magnetic field. The effective value extracted from the MBN signal is referred to as *MBN_rms_*. MBN was measured along the grinding direction (GD—along the length of the samples) as well as in the transversal direction TD. MBN envelopes are plotted as the short-time root mean square values of the MBN signal.

The depth profiles of residual stress as well as dislocation density were measured using the XRD technique (Proto iXRD Combo diffractometer (Proto Manufacturing Inc., Oldcastle, ON, Canada) using CrKα radiation, the average effective penetration depth ~ 5 μm, scanning angle ± 39°, Bragg angle 156.4°). The residual stress was calculated from shifts in the 211 reflection. The Winholtz and Cohen method and X-ray elastic constants ½*S*_2_ = 5.75 TPa^−1^ and *S*_1_ = −1.25 TPa^−1^ were applied. Removal of the subsequent layer before XRD measurement was carried out by electrochemical polishing. Residual stresses were also measured in GD and TD. Dislocation density *δ* was calculated on the basis of the XRD peak width (*FWHM*—full width at half maximum) as follows:

(1) $$\delta =\;\frac{1}{D^{2}}\;\;\;\;\;\;\;\;\;\;(m^{-2})$$

where *D* is crystallite size calculated via (2)

(2) $$D=\frac{K\cdot \lambda }{\beta \cdot \cos\theta }\;\;\;\;\;\;\;(\mathrm{nm})$$

where *K* is the constant (0.89), *λ* is the X-ray wavelength (2.291 Å), *β* = *FWHM* and *θ* is equal to the Bragg angle mentioned above.

The thermal softening initiated by the grinding was analysed via the metallographic images on the small specimens of length 10 mm cut on Secotom 50 along GD (Struers ApS, Ballerup, Denmark). The specimens were routinely hot moulded, ground, polished and etched by 3% Nital etchant for 8 s. Metallographic observations were performed on the light microscope Leica (Wetzlar, Germany) with the Z-stack function. Finally, the thermal softening was also analysed via the microhardness profiles measurements *HV0.05* using Innova Test 400TM (INNOVATEST Europe BV, Maastricht, The Netherlands) on the hot moulded specimens after the metallographic observations.

**Figure 1:**
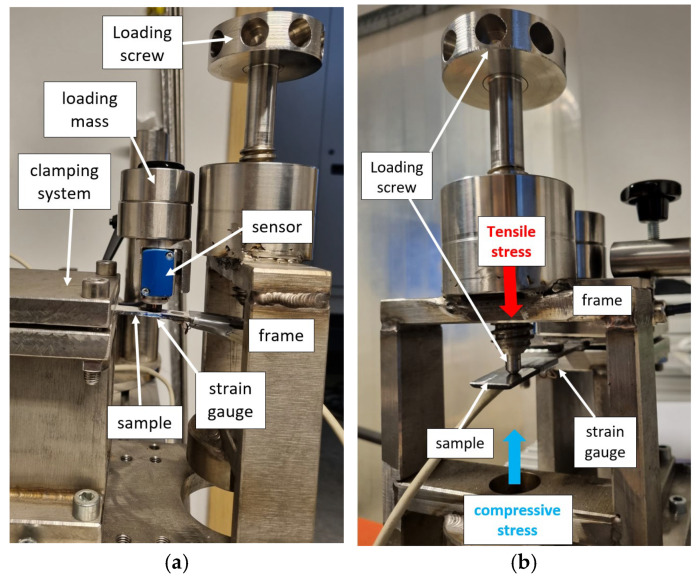
Sample clamping and bending in the self-made device. (**a**) Side view, (**b**) front view.

Sensitivity of MBN against the external loading was carried out via the bending test in order to investigate the tensile as well as compressive regions. The magnitude of residual stresses was ±600 MPa with a step of 50 MPa. Such a magnitude of the assessed stress more or less corresponds to the maximum magnitude of the tensile residual stresses found on the surface of 100Cr6 as a result of the growing grinding wheel wear reported earlier [12]. The sample bending was performed on the self-made device, especially developed for this purpose; see Figure 1. The sample was clamped into the clamping region and fastened with the screws. The stress amplitude was measured on the opposite side of the sample in the region of MBN measurement with respect to the distance from the clamping by the strain gauge HBM 6/120 LY11 (resistance 120 Ω ± 0.35% and *k*-factor 2.09 ± 1.0%, Hottinger Baldwin Messtechnik GmbH (HBM), Darmstadt, Germany). Information about the stress state was mediated via DasyLab2016 software 14.2.0 (A/D conversion via the NIcDAQ-9714 motherboard equipped with the NI9237 module, National Instruments (NI)/Emerson, Austin, TX, USA). The load was developed manually via the loading screw. The MBN sensor was loaded with a mass of 750 g to avoid metastable data reading (excessive mechanical vibration in the sensor-sample interface).

In order to investigate the contribution of stress state on MBN, the directional magnetostriction along GD *λ_GD_* as well as TD *λ_TD_* were measured. The volumetric one *λ_S_* can then be calculated as follows:

*λ*_*S*_ = 2/3 (*λ*_*GD*_ − *λ*_*TD*_) (3)

The magnetostriction measurements were carried out on the small specimen of size 35 × 20 × 3 mm (surface without grinding—as received after heat treatment). These specimens were equipped with the strain gauges 1-XY11-3/120 (two perpendicular nets for the simultaneous measurement along two perpendicular directions, Hottinger Baldwin Messtechnik GmbH (HBM), Darmstadt, Germany). The specimen elongation along the increasing magnetic field was measured in the Phylatex Physic Gerate system (PPG, Frankenberg, Germany), generating the uniaxial magnetic field controlled by the voltage source XG100-15 (Sorensen, San Diego, CA, USA). The Tektronix A622 AC/DC current probe provided information about the magnetic field (Tektronix, Inc., Beaverton, OR, USA). The measured signals (with respect to magnetostriction as well as magnetising field) were stored in DasyLab 2016 (sampling frequency 1 kHz). The analogue signal was A/D converted by the use of the NIcDAQ-9714 motherboard equipped with the NI9237 module for the strain gauge data and the NI9215 module for the magnetising field data.

## 3. Results of Experiments and Their Discussion

**Figure 2:**
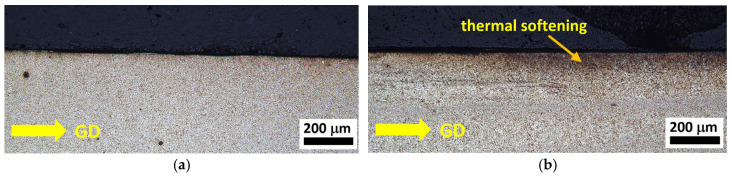
Metallographic images of the surface after grinding. (**a**) *a_p_* = 0.01 mm, (**b**) *a_p_* = 0.03 mm.

Figure 2 depicts the metallographic images of the ground surfaces along GD as a function of the infeed rate. In the case of the low infeed rate (0.01 mm), the ground surface is mostly free of thermally softened regions. However, the dark zone of quite deep extent towards the depth can be found for the infeed rate of 0.03 mm. This thermally softened region appears dark due to the lower dislocation density and therefore reduced resistance against the etchant. It should also be noted that a certain surface layer affected by the grinding was removed during the final stage of grinding—the spark-out passes.

The grinding process can suffer from excessive heat generation, especially under the higher removal rates [1,2]. This heat generation is a result of high cutting speeds. The unfavourable heat partitioning, when approx. 90% of this heat penetrates to the workpiece, which is due to the low thermal conductivity of the Al_2_O_3_ wheel as well as the low feed rates. For these reasons, ground surfaces should be monitored in a proper manner in order to avoid the usage of those components being thermally damaged.

**Figure 3:**
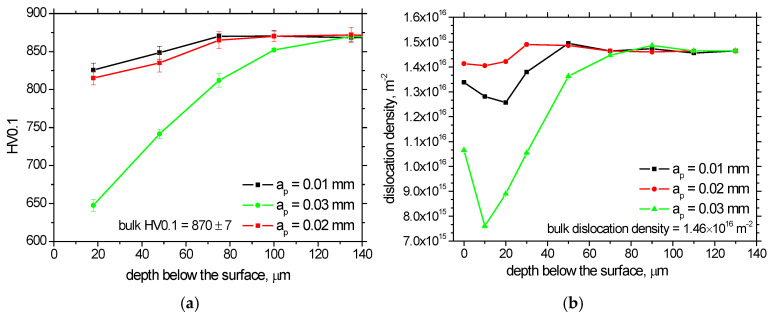
*HV0.1* and dislocation density *δ* depth profiles. (**a**) *HV0.1*, (**b**) dislocation density *δ*. Note: the uncertainties with respect to *δ* oscillate in the range of 2.03 × 10^12^ up to 1.68 × 10^13^ m^−2^.

The thermally initiated softening reduces the dislocation density in the near-surface region, and the penetration depth of this effect depends on the thermal conductivity of the counter parts, grinding conditions, as well as the degree of the grinding wheel wear [19,20]. Apart from the metallographic observations, thermal softening in this particular case can also be demonstrated by the microhardness depth profiles (see Figure 3a) as well as the dislocation density; see Figure 3b [21]. Figure 3 clearly demonstrates the remarkable decrease in *HV0.1*, as well as the dislocation density for the infeed 0.03 mm, as well as their deeper extent, as contrasted against the lower infeed rates.

**Figure 4:**
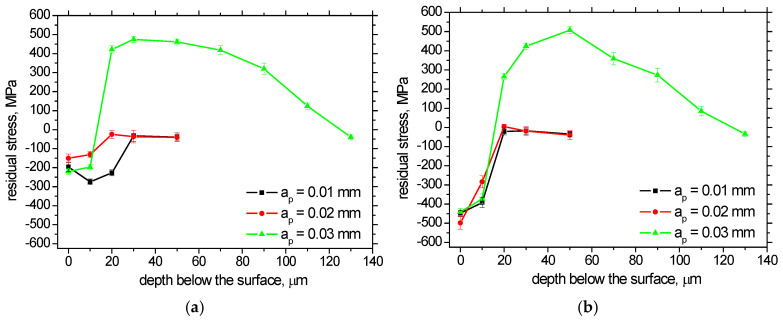
Residual stress depth profiles. (**a**) GD, (**b**) TD.

The remarkable differences among the samples can also be demonstrated by the residual stress depth profiles; see Figure 4. The following findings can be reported:

- The surface residual stresses for the different infeed rates are quite similar;
- The amplitude of these surface residual stresses in TD is more as contrasted against GD;
- The depth profiles of residual stresses for the infeed rates of 0.01 mm and 0.02 mm are quite similar (their penetration depth is quite low);
- The residual stresses in the surface as well as sub-surface regions are compressive;
- The residual stresses in the deeper layers within the MBN sensing depth (about 40 μm) for the infeed rate of 0.03 mm are the tensile ones and penetrate to a depth up to 130 μm, as compared with the lower infeed rates.

**Figure 5:**
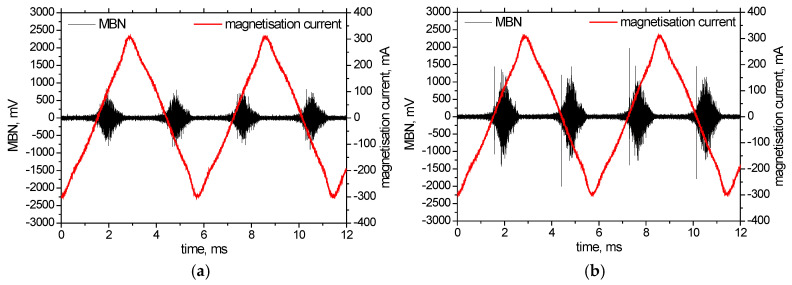
MBN filtered signals in GD. (**a**) *a_p_* = 0.01 mm, (**b**) *a_p_* = 0.03 mm.

**Figure 6:**
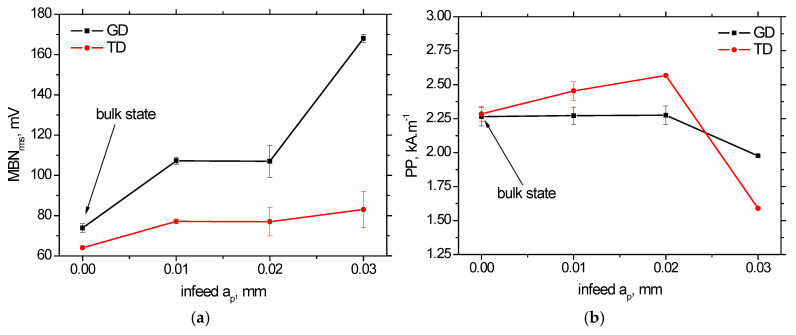
*MBN_rms_* and *PP* versus infeed rate *a_p_*. (**a**) *MBN_rms_* versus *a_p_*, (**b**) *PP* versus *a_p_*.

The thermal softening and the corresponding decrease in the reduced dislocation density take the major role in the evolution of MBN along the infeed rate; see Figure 5 and Figure 6. It has already been investigated that the bearing steel 100Cr6 contains large carbides, which are quite stable against the thermal load during grinding [12]. Furthermore, the retained austenite fraction is nearly unaffected by grinding [12], whereas the dislocation annihilation and the corresponding drop of dislocation density can be found (as depicted in Figure 3b). The weaker opposition of the matrix containing the reduced dislocation density against DWs in motion explains the higher *MBN_rms_*, especially in the case of the infeed rate of 0.03 mm. Interpretation of *MBN_rms_* in terms of the residual stresses is quite controversial. On one hand, the tensile stresses in the sub-surface region for the infeed rate of 0.03 mm might contribute to the higher *MBN_rms_*. The lower *MBN_rms_* in TD might be explained by the higher amplitude of compressive stresses; see Figure 4 and Figure 6. On the other hand, this effect has only the complementary influence since the removal of compressive residual stresses initiated by the grinding in the case of all infeed rates results in a decrease in *MBN_rms_* (compare *MBN_rms_* in GD and TD for the bulk and after the grinding in Figure 6). Also, the previous study [12] in this field clearly demonstrated that no DW realignment can be found and DWs orientation is more or less isotropic.

The theory about the thermal softening also confirms the evolution of *PP* extracted from the MBN envelopes (see Figure 6). The *PP* in GD remains fully unaffected at the lower infeed rates, followed by the remarkable decrease for the infeed rate of 0.03 mm. It has already been reported that this parameter is closely connected with the magnetic as well as mechanical hardness [22,23]. The certain growth of *PP* in TD might be linked with the constrained plastic deformation of the near-surface and preferential orientation of the ground matrix [24]. Furthermore, the role of the initial matrix anisotropy due to technological inheritance should be considered as well when the *MBN_rms_* for the bulk state in GD is more than that for TD; see Figure 6.

As noted earlier, the influence of residual stress on MBN in the case of bearing steel is quite limited and the role of microstructure prevails. This finding is fully in agreement with the Neel theory [13] postulating that coercive force and corresponding DWs motion during magnetisation is mostly driven by the presence (and density) of lattice imperfections as contrasted against the internal stress fields.

Investigating the role of residual stresses and microstructure (expressed in terms of dislocation density in this particular case) on MBN, one might overlook the different sensing depth of MBN and XRD. While the estimated reading depth of MBN in this particular case is about 50 μm [25], the XRD penetration depth is only 5 μm. For this reason, the effective sum of residual stresses (*SERS*) and dislocation density (*SEDD*) can be calculated as follows:

(4) $$SERS=\;\sum_{i=1}^{4}k_{i}({RS}_{i}-\;{RS}_{i-1}-{RS}_{B})(t_{i}-\;t_{i-1}),$$

(5) $$SEDD=\sum_{i=1}^{4}k_{i}({DD}_{i}-{DD}_{i-1}-{DD}_{B})(t_{i}-t_{i-1}),$$

where

- The depth of 50 μm is divided into four layers of a thickness of 12.5 μm;
- *k_i_* is the weighing constant taking into consideration MBN pulses attenuation towards the depth (*k*_1_ = 0.5, *k*_2_ = 0.25, *k*_3_ = 0.145 and *k*_4_ = 0.105) following the MBN signal damping [26,27];
- *RS_i_* − *Rs_i_*_−1_ (*DD_i_* − *DD_i_*_−1_) represents the average residual stress (dislocation density) within each layer of the thickness *t_i_ − t_i_*_−1_, which is constant (12.5 μm);
- *RS_B_* is the bulk stress, and *DD_B_* is the bulk dislocation density.

**Figure 7:**
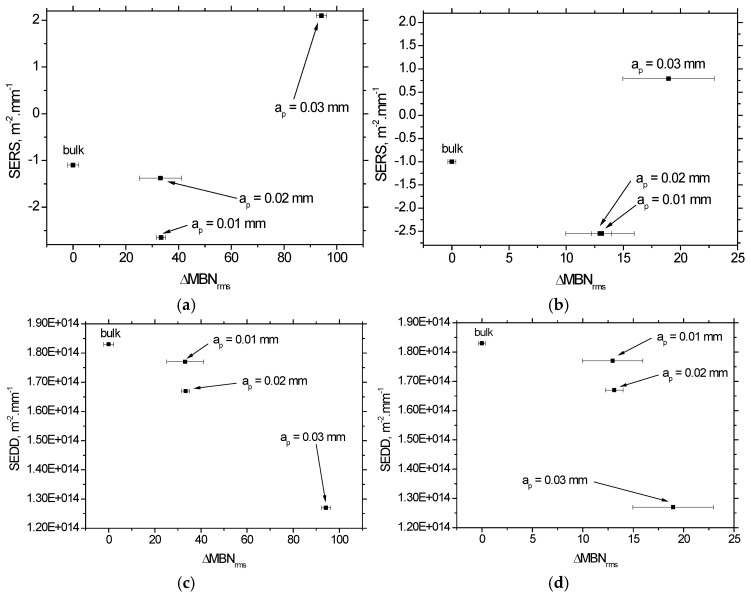
*SERS* and *SEDD* versus ∆*MBN_rms_*. (**a**) *SERS* versus ∆*MBN_rms_* in GD, (**b**) *SERS* versus ∆*MBN_rms_* in TD, (**c**) *SEDD* versus ∆*MBN_rms_* in GD, (**d**) *SEDD* versus ∆*MBN_rms_* in TD.

Figure 7a,b clearly depicts that the role of residual stress is only minor when the positive ∆*MBN_rms_* for *a_p_* = 0.01 and 0.02 mm are linked with the more negative *SERS*. Only in the case of *a_p_* = 0.03 mm, the synergistic contribution of residual stress on MBN can be considered. Furthermore, when the role of stress state is considered, DWs realignment should increase the MBN in one direction at the expense of the perpendicular one [11], but it does not (see Figure 6a). On the other hand, the continuous growth of MBN can be linked with the decreasing dislocation density, as depicted in Figure 7c,d.

**Figure 8:**
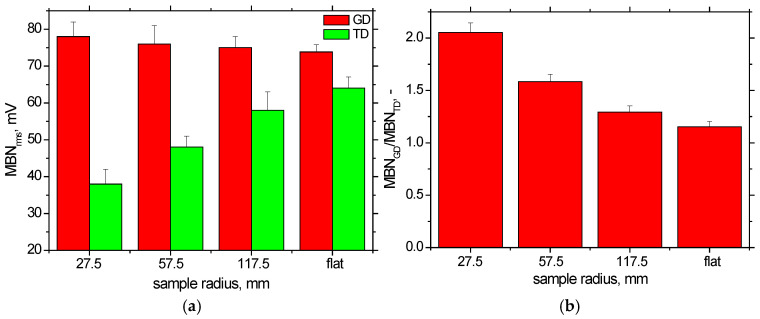
*MBN_rms_* and *MBN_GD_*/*MBN_TD_* as a function of sample geometry after heat treatment. (**a**) *MBN_rms_* as a function of sample geometry, (**b**) *MBN_GD_*/*MBN_TD_* as a function of sample geometry.

The flat geometry of the employed samples plays an important role. Investigation of the magnetic anisotropy on the real bearing components is a debatable issue since *MBN_rms_* in the different directions are different as a result of matrix anisotropy, as well as the superimposing contribution of the different sample magnetisation due to the complex sample’s curvature; see Figure 8. Especially, MBN in TD is remarkably affected by the sample curvature (radius, see Figure 8a), which in turn results in the different *MBN_GD_*/*MBN_TD_*; see Figure 8b. Having the flat samples, the shape effect does not take a strong role, and the contribution of matrix anisotropy can be singled out.

**Figure 9:**
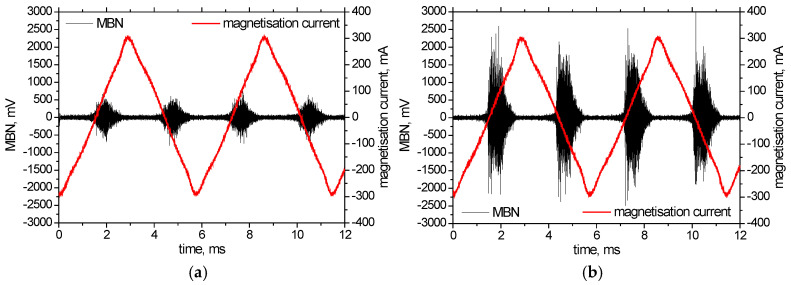
MBN filtered signals in GD and *a_p_* = 0.03 mm. (**a**) −600 MPa, (**b**) +600 MPa.

**Figure 10:**
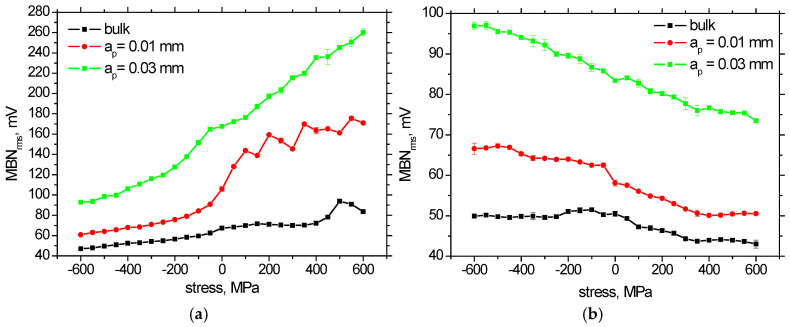
*MBN_rms_* versus exerted stress. (**a**) GD, (**b**) TD.

The influence of external stress on MBN might be different. The realignment of DWs due to the presence of the different amplitudes of external stress as well as its direction is clear; see Figure 9 and Figure 10. The *MBN_rms_* is growing along with the tensile stresses and drops down along with the compressive ones when the magnetisation field is loaded along the direction of the exerted field (linked with GD). This evolution is fully reversed when the altering magnetising field is along TD; see Figure 10. The better sensitivity against stress can be obtained in the thermally softened surfaces. These stress versus *MBN_rms_* evolutions (especially for the infeed rate 0.03 mm) are shifted into the higher *MBN_rms_*, and the sensitivity is better due to the easier DWs motion, as well as the considered higher fraction of unpinned DWs contributing to the MBN. Figure 10 clearly indicates that the MBN in GD increases from the compressive towards the tensile stresses at the expense of reduced MBN in TD. However, the increase in *MBN_rms_* in GD is much stronger as contrasted against the TD; see Figure 10.

**Figure 11:**
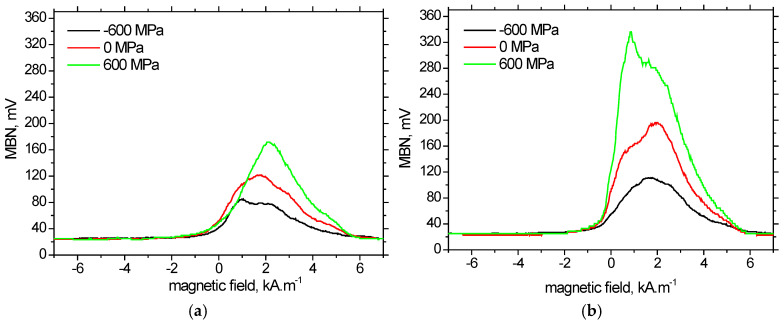
MBN envelopes in GD. (**a**) Bulk state, (**b**) *a_p_* = 0.01 mm.

**Figure 12:**
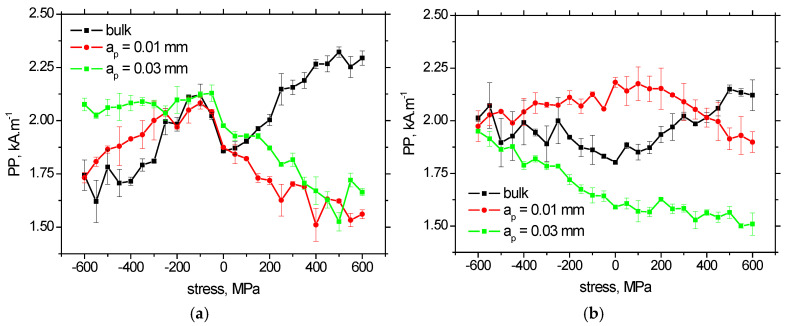
*PP* versus exerted stress. (**a**) GD, (**b**) TD.

The extracted MBN envelopes (see Figure 11) demonstrate that the *PP*s (obtained from the MBN envelopes) are altered with respect to the external stress (see also Figure 12). However, the evolution of *PP* versus stress is not monotonic and straightforward, as contrasted with the evolution of *MBN_rms_*.

The *PP* in GD exhibits the local maxima (or the global ones in the case of ground surfaces). The *PP* is dropping down along with the increasing amplitude of external stresses (tensile as well as compressive ones) for the ground surface, as contrasted against the bulk state. Also, the evolution of *PP* for bulk states in TD is quite different, especially in the region of tensile stresses. Explanation of such behaviour is still a challenging task, and further investigation should be carried out in this field. However, it is considered that the presence of residual stresses of variable amplitude as well as directions (as a function of the infeed rate) might have only a superimposing role.

**Figure 13:**
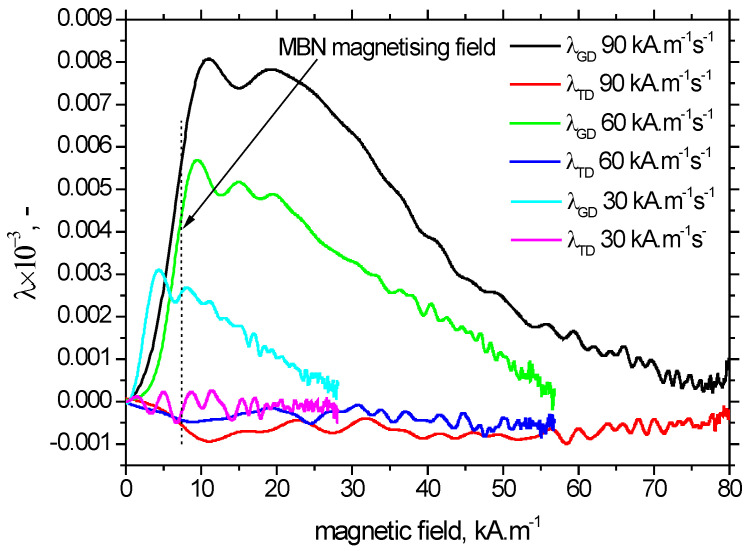
Evolution of λ in RD and TD with magnetic field as a function of magnetising field rate change.

**Figure 14:**
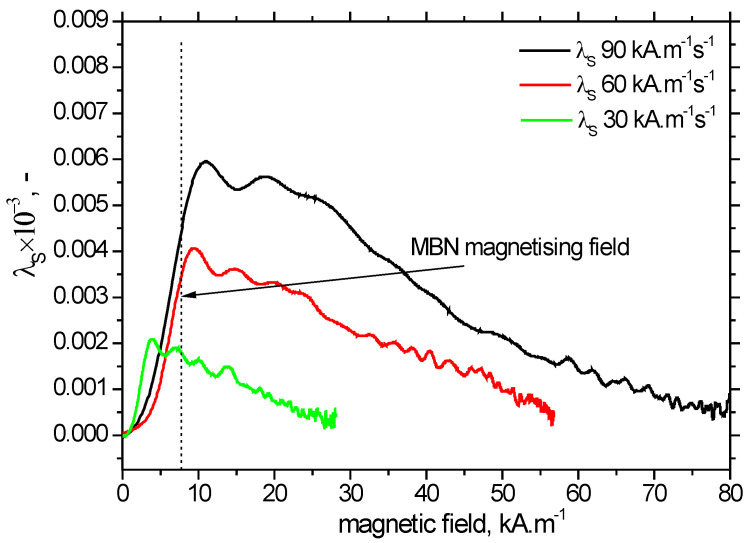
Evolution of λ_S_ with magnetic field as a function of magnetising field rate change.

The sensitivity of *MBN_rms_* against the exerted stress is much better compared with *PP*, and the non-systematic evolution of *PP* discriminates this MBN feature as a suitable parameter for the non-destructive assessment of stress state in this kind of bearing steels. This finding also proves the measured magnetostriction depicted in Figure 13 and Figure 14. λ_S_ as well as *λ_GD_* are quite weak (as contrasted against, for example, Ni [8,28]). The positive *λ_GD_* can be found at the expense of dropping λ_TD,_ which corresponds to the ascending *MBN_rms_* in GD (see Figure 10a) and the descending *MBN_rms_* along TD (see Figure 10b). The valuable lower *λ_TD_* also explains much lower descend (expressed in terms of *MBN_rms_*) as compared with the much stronger ascend of *MBN_rms_* along GD linked with much stronger *λ_GD_*.

**Figure 15:**
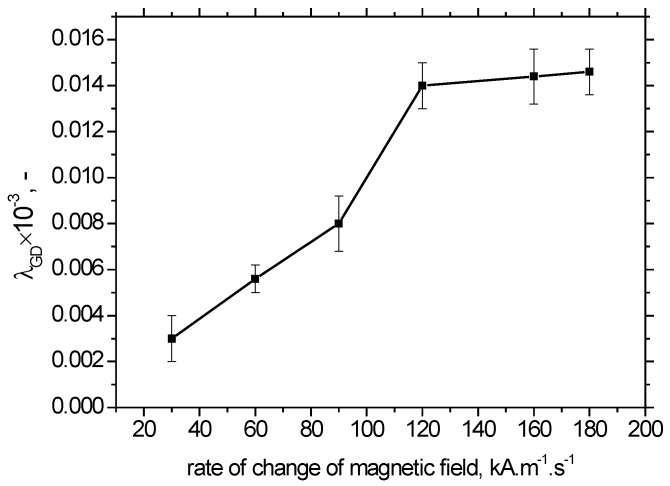
Evolution of *λ_GD_* along with the rate of change in magnetising field.

One interesting aspect can be reported with respect to measured *λ_GD_* and *λ_TD_*. These features are sensitive to the rate of change in the magnetising field. The lower rate of change results in the lower *λ_S_*, *λ_TD_* and *λ_GD_* and vice versa; see Figure 13 and Figure 14. The rate of change in magnetising field during the MBN measurement is about 1 order more (≈610 kA·m^−1^·s^−1^) than that during the magnetostriction measurements. However, Figure 15 illustrates that the *λ_GD_* growth saturates beyond 120 kA·m^−1^·s^−1^. Therefore, the true *λ_GD_* during the MBN measurement in this particular case can be about 0.0075 × 10^−3^.

## 4. Conclusions

This study investigates the role of stress state as well as dislocation density on MBN after grinding bearing steel 100Cr6 as a function of the infeed rate and the corresponding thermal load of the ground surface. The study also analyses the evolution of MBN along with the elastic external stresses with respect to the magnetostriction. The main findings of this study can be summarised as follows:

- The higher infeed rates are linked with the stronger thermal softening in grinding;
- This thermal softening is linked with the remarkable *MBN_rms_* due to the decreasing dislocation density, whereas the role of residual stress state is only minor;
- The external compressive stresses decrease *MBN_rms_*, whereas the tensile stresses make *MBN_rms_* stronger when the direction of the altered magnetic field is along the direction of the exerted stress;
- This evolution is fully reversed when the direction of the magnetising field is perpendicular to the stress;
- The *PP* exhibits the non-systematic behaviour in this particular case;
- The positive magnetostriction along GD *λ_GD_* corresponds to the increasing *MBN_rms_* along the tensile stresses, whereas the negative *λ_TD_* along TD results in the descending *MBN_rms_*.

## Data Availability

The data presented in this study are available on request from the corresponding author due to the raw data required to reproduce these findings cannot be shared easily due to technical limitations (some files are too large).
